# Ketogenic effect of coconut oil in ALS patients

**DOI:** 10.3389/fnut.2024.1429498

**Published:** 2024-07-17

**Authors:** Sandra Carrera-Juliá, Elena Obrador, Rafael López-Blanch, María Oriol-Caballo, Paz Moreno-Murciano, José M. Estrela

**Affiliations:** ^1^Department of Nutrition and Dietetics, Catholic University of Valencia San Vicente Mártir, Valencia, Spain; ^2^Department of Physiology, University of Valencia, Valencia, Spain; ^3^Scientia BioTech, Valencia, Spain

**Keywords:** amyotrophic lateral sclerosis, nicotinamide riboside, pterostilbene, coconut oil, ketogenic diet

## Abstract

A recent pilot study in amyotrophic lateral sclerosis (ALS) patients analyzed the effect of a Mediterranean diet (MeDi) supplemented with nicotinamide riboside (NR, a NAD^+^ promoter), pterostilbene (PTER, a natural antioxidant) and/or coconut oil on anthropometric variables in ALS patients. The results suggested that the MeDi supplemented with NR, PTER and coconut oil is the nutritional intervention showing the greatest benefits at anthropometric levels. Over the last 30 years, glucose intolerance has been reported in ALS patients. Thus, suggesting that an alternative source of energy may be preferential for motor neurons to survive. Ketone bodies (KBs), provided through a MeDi with a lower carbohydrate content but enriched with medium chain triglycerides, could be a therapeutic alternative to improve the neuromotor alterations associated with the disease. Nevertheless, the use of a coconut oil-supplemented diet, as potentially ketogenic, is a matter of controversy. In the present report we show that a MeDi supplemented with coconut oil increases the levels of circulating KBs in ALS patients.

## 1 Introduction

Amyotrophic lateral sclerosis (ALS) is a nervous system disease that affects nerve cells in the brain and spinal cord. ALS damages motor neurons (MNs) and causes loss of muscle control. In a recent report, we showed that a Mediterranean Diet (MeDi) complemented with nicotinamide riboside (NR, a NAD^+^ promoter) and pterostilbene (PTER, a natural polyphenol and antioxidant), and the MeDi enriched with coconut oil are dietary interventions that render benefits at anthropometric levels (1).

Whether supplementation of the MeDi diet with coconut oil may be considered a ketogenic diet (KetoDi) is controversial. Norgren et al. (2) felt surprised because one of his works (3) was used in Carrera-Juliá et al. (1) to support the claim that “nutritional supplementation with coconut oil could be a good way to promote the synthesis of ketone bodies (KBs).” That quote was a mistake on our part.

In Norgren et al. (3), the study showed that after consuming 30 grams of coconut oil, circulating levels of β-hydroxybutyrate (BHB) were not higher compared to those after consuming sunflower oil, which contains no medium-chain triglycerides and was used as the control. Additionally, combining coconut oil with carbohydrates did not increase BHB levels. According to several publications since 2017, Norgren et al. (2) argues that only C8 has a significant ketogenic effect, unlike C10 and C12 (3–6). In coconut oil, C12 makes up about half of the fatty acid content, while C8 is only 7% (7). Thus, Norgren et al. (2) conclude that even with a daily dose of 60 g [as used in our study (1)], coconut oil may not induce substantial ketosis, as the C8 content amounts to only about 4 grams. Following this line of reasoning, in our study’s approach of five meals per day and a carbohydrate target of 40% for the “ketogenic” MeDi (1), it is unlikely that BHB levels would exceed 0.1 mmol/L (8). They also note that a typical KetoDi should have a carbohydrate content of 10% or less (2).

In the present report we have tried to clarify this problem, but specifically in relation to ALS patients who received a MeDi supplemented with coconut oil.

## 2 Materials and methods

### 2.1 Study design and eligibility

Patients were recruited thanks to different associations of ALS patients. Eligible participants were required to be 40 years of age or older. All ALS patients were diagnosed with probable or definite (sporadic) ALS by El Escorial criteria, having experienced spinal symptoms for over 6 months. The study included both female and male participants; female participants were not lactating, tested negative for pregnancy, and consented to use effective contraception for the study’s duration. All patients were treated with riluzole in the standard prescribed dosage.

We excluded ALS patients that met the following criteria: tracheostomy, invasive ventilation, or non-invasive positive pressure ventilation; gastrostomy; evidence of major psychiatric disorder or clinically evident dementia; diagnosis of a neurodegenerative disease in addition to ALS; are taking current medication apart from riluzole that, in the opinion of their neurologists, would make the patient unsuitable for study participation; have a recent history (within the previous 6 months) or current evidence of alcohol or drug abuse; have concurrent unstable disease involving any system (e.g., carcinoma other than basal cell carcinoma), any cardiac dysrhythmia, myocardial infarction, clinical or ECG signs of myocardial ischemia, cardiac insufficiency, angina symptoms, current symptoms of coronary artery disease; have a baseline QTc (Bazett) > 450 msec for males and > 470 msec for females; patients with known hepatitis B/C or HIV positive serology; have renal impairment defined as blood creatinine >2 × ULN (upper limit of normal); have hepatic impairment and/or liver enzymes (ALAT or ASAT) > 3 × ULN; hemostasis disorders or current treatment with oral anticoagulants; have participated in any other investigational drug or therapy study with a non-approved medication, within the previous 3 months.

### 2.2 Ethics statement

This study received approval from the University of Valencia Institutional Review Board on Human Studies, and all participant-related procedures were sanctioned by the University of Valencia Ethics Committee, under the reference number H1479983999044. All interventions conducted adhered to the guidelines set forth in the Declaration of Helsinki (9). Participants were given a written informed consent form, which they signed after being fully informed about the study’s procedures and its nature.

### 2.3 Diet

Healthy and ALS control participants consumed a Mediterranean-style diet (approximately 2,300 kcal/day; 55% carbohydrates, 30% fat, 15% proteins) [refer to, e.g., Davis et al. (10)]. This MeDi was structured into five meals daily. The diet featured slow-releasing carbohydrates. Vitamin and mineral levels were tailored to meet the recommended dietary allowances set by the EFSA (European Food Safety Authority). The MeDi enriched with coconut oil (approximately 2,300 kcal/day; 40% carbohydrates, 40% fat, 20% proteins) incorporated a total of 60 mL of coconut oil (Herbalist Navarro, Valencia, Spain), where 30 mL were given at breakfast and 30 mL at dinner.

### 2.4 Nicotinamide riboside and pterostilbene administration

Treated participants, healthy subjects or ALS patients, received 15 mg NR and 2.5 mg PTER/kg body weight/day (administered orally in standard gelatin capsules), with half of the dosage given in the morning (30 min before breakfast) and the other half in the evening (30 min before dinner).

### 2.5 ALSFRS-R test

The revised ALS functional rating scale (ALSFRS-R) test (11) was applied to the ALS group at baseline and 2 months after the intervention.

### 2.6 Anthropometric assessment

The assessment encompassed body weight, skinfold measurements (triceps, subscapular, iliac crest, and abdominal), body circumferences (waist and hip), and small bone diameters (humerus, bistyloid, femur). Body Mass Index (BMI), Waist-Hip Index (WHI), and Waist-Height Ratio (WHR) were also calculated. Muscle weight was determined using the Matiegka formula (12) to estimate the percentage of muscle mass. All these procedures were executed as detailed previously (1).

### 2.7 Monitoring

Each patient received a tailored dietary plan and nutritional supplements, accompanied by personalized dietary advice aimed at enhancing their eating habits. Following the initiation of the intervention, individualized follow-up phone calls were conducted to discuss any concerns, including potential difficulties with swallowing, changes in taste, or texture tolerance. These monitoring sessions facilitated necessary adjustments to the dietary plan to promote adherence.

### 2.8 Statistical analysis

Data are reported as mean values ± SD. Analysis was conducted using a two-way analysis of variance (ANOVA) (SPSS Statistics 29 for Windows; SPSS Inc., Chicago, IL, United States). Variance homogeneity was assessed using the Levene test. The null hypothesis was accepted for all values where the *F* value was not significant, with *p* > 0.05. For data with a significant F value, further examination was performed using the Tukey’s test at *p* < 0.05.

## 3 Results and discussion

Healthy controls and ALS patients were fed a MeDi × 4 weeks before adding the supplements [NR, PTER and coconut oil (60 g/day) as in Carrera-Juliá et al. (1)]. All participants were evaluated after that 4-weeks initial period (T0) and 2 months after being fed with the MeDi diet supplemented with NR + PT + coconut oil (2 mo.) (Table 1).

**Table 1:** Effect of a MeDi diet supplemented with nicotinamide riboside, pterostilbene and coconut oil on anthropometric parameters in healthy controls and ALS patients.

	Healthy controls		ALS patients	
	MeDi (T_0_)	MeDi + NR + PT + Coco oil (2 mo.)	MeDi (T_0_)	MeDi + NR + PT + Coco oil (2 mo.)
Male	10		12	
Female	7		9	
Age (years)	54.5 ± 5.9		55.7 ± 4.3	
Weight (kg)	70.5 ± 4.9	69.8 ± 7.6	69.4 ± 5.7	71.3 ± 8.3
BMI (kg/m2)	23.5 ± 3.9	24.3 ± 6.0	23.9 ± 2.6	25.8 ± 3.3
Fat mass (%)	20.0 ± 4.1	26.1 ± 5.2	21.6 ± 3.0	22.17 ± 2.9
Muscle mass (%)	31.0 ± 3.3	28.0 ± 4.5	29.3 ± 3.5	31.5 ± 3.0

A two-way analysis of variance (ANOVA) was used to make comparisons among the different groups., although no significant differences were found. All participants were fed the same MeDi diet [as in Carrera-Juliá et al. (1)] 4 weeks before adding the supplements. T0 (time cero, pre-intervention assessment), 2 mo. (post-intervention assessment at 2 months).

We observed that ALS patients fed the supplemented MeDi diet, but not the healthy controls, showed higher levels of fasting glucose (Table 1). A fact which seems coherent with the impairment of glucose utilization that ALS patients develop as the disease progresses (13, 14). Indeed, there has been a documented decrease in glucose absorption in the spinal cord and various cerebral areas, including the motor, frontal, and occipital cortices, among both human patients and animal models of ALS. This is associated with diminished glucose consumption and functional alterations in these areas [as recently discussed by Tefera et al. (15)]. This implies a potential and progressive reduction in the capacity of MNs to generate energy from glucose, a metabolic issue that does not affect, for example, neighboring astrocytes as demonstrated in the SOD1^G93A^ ALS murine model (16). If obtaining energy from glucose progressively fails, it is reasonable to infer that MNs might switch to an alternative energy source for survival. Based on this assumption, research on SOD1^G93A^ mice indicates that a KetoDi could slow the progression of ALS’s clinical and biological signs (17). A KetoDi enhances the production of KBs, compounds that neurons can utilize as an energy source in the brain (18). Since KBs can exert neuroprotective effects, it has been suggested that inducing moderate ketosis through regular consumption of medium-chain fatty acids could be beneficial (17, 19).

Interestingly, a high intake of polyunsaturated fatty acids and vitamin E is linked to a 50–60% lower risk of developing ALS, with these nutrients appearing to work synergistically (20). Furthermore, a study tracking 1,002,082 individuals, of which 995 developed ALS, found that increased intake of ω-3 polyunsaturated fatty acids correlated with a lower ALS risk (21). It has also been observed that the synthesis of BHB can reduce mitochondrial production of reactive oxygen species in stressed neurons by promoting NADH oxidation (22), thus reducing oxidative stress. This oxidation, and the resultant higher NAD^+^/NADH ratio, play crucial roles in cellular redox balance and the activation of protein deacetylases like sirtuins 1 and 3 (23). Mechanisms which are involved in the pathophysiology of ALS (24). Moreover, KBs can (a) enhance mitochondrial biogenesis and improve mitochondrial efficiency; (b) lower inflammation by reducing pro-inflammatory cytokines (e.g., TNF-α, IL-1β); (c) increase the levels of BDNF, which supports neuron survival and growth; (d) enhance autophagy, a process that clears damaged cellular components, potentially protecting against neurodegeneration; (e) modulate glutamate levels, reducing excitotoxicity that contributes to motor neuron death; (f) restore myelin sheaths of neurons; (g) modulate the gut microbiota; and (h) enhance dopamine production and increase conversion of glutamine to GABA (17, 25–28). All these mechanisms are potentially neuroprotective.

Consequently, potential KetoDi-related biomarkers may include (but not limited to): (a) elevated levels of BHB and acetoacetate (AA) in blood and cerebrospinal fluid (CSF), which indicate ketosis; (b) improved ATP production, a marker of enhanced mitochondrial function; (c) increased cytochrome c oxidase activity, which indicates better mitochondrial respiratory chain function; (d) reduced levels of isoprostanes, protein carbonyls and/or 8-hydroxy-2′-deoxyguanosine, which reflect oxidation of lipids, proteins and DNA, respectively; (e) elevated superoxide dismutase activity, which indicates improved antioxidant defense; (f) lower levels of C reactive protein and pro-inflammatory cytokines (e.g., TNF-α, IL-1β, and IL-6); (g) elevated BDNG, which associates with neuroprotection and neuronal health (18, 29, 30).

Nevertheless, up to now, a KetoDi for ALS cannot not be recommended since regular clinical trials still are pending (31).

**Figure 1:**
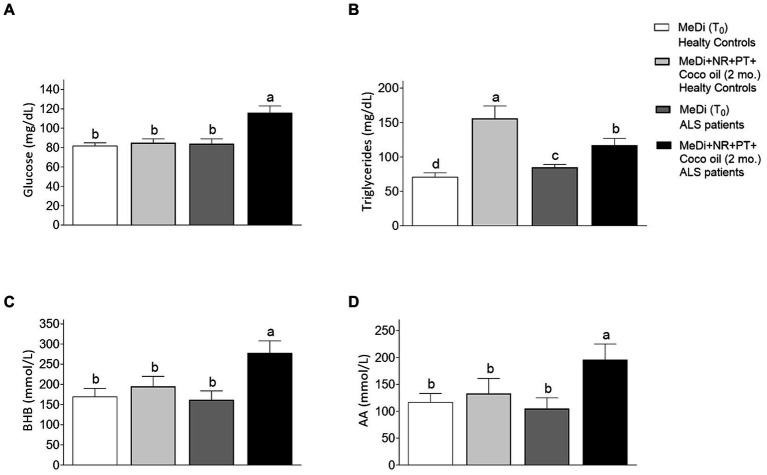
Effect of a MeDi diet supplemented with nicotinamide riboside, pterostilbene and coconut oil on **(A)** glucose, **(B)** triglycerides, **(C)** beta-OH-butyrate (BHB), and **(D)** acetoacetate (AA) in healthy controls and ALS patients. A two-way analysis of variance (ANOVA) was used to make comparisons among the different groups. Different letters indicate differences *p* < 0.05. The letter “a” is given to the highest mean value, and the letter c to the lowest. Data under “b” is significantly different from “a.” Data under “c” is significantly different from “a and b.” ALSFRS-R of the ALS patients at T_0_: 40.3 ± 5.2. Glucose, triglycerides, BHB and AA were measured at 8.00 am before breakfast. All participants were fed the same MeDi diet [as in Carrera-Juliá et al. (1)] 4 weeks before adding the supplements. T_0_ (time cero, pre-intervention assessment), 2 mo. (post-intervention assessment at 2 months).

As shown in Table 1, we found no significant differences in anthropometric parameters comparing healthy controls and ALS patients, fed a MeDi diet or a MeDi diet supplemented with NR, PTER and coconut oil. No significant differences were found in plasma KB levels comparing healthy controls fed a MeDi or after being fed a MeDi+NR + PT + Coconut oil (Figures 1C,D). A fact coherent with the observations of Norgren et al. and others (see above). However, triglyceride levels (Figure 1B) were lower, and KBs (Figures 1C,D) and glycemia (Figure 1A), higher in ALS patients fed with MeDi+NR + PT + Coconut oil as compared to healthy controls fed the same supplemented diet. These differences must necessarily depend on the coconut oil and on the fact that ALS patients may use more fatty acids than healthy controls as an alternative source of energy. Therefore, although the use of a coconut oil-supplemented diet cannot be defined as a “KetoDi,” it is also evident that a coconut oil supplemented-MeDi diet can increase KBs in the plasma of ALS patients.

The % of carbohydrates in a diet conditions the use of fatty acids as a source of energy. However, it is important to note that during periods of rest, our skeletal muscle cells, which drive human movement, predominantly rely on fats as their primary fuel source. They switch to glucose utilization only when insulin levels increase, typically in response to elevated blood sugar levels requiring neutralization and storage as glycogen or fat (32). This physiological feature is relevant for patients with reduced mobility, as it occurs in ALS. Although there is not one “standard” KetoDi with a specific ratio of macronutrients, based on different sources, a KetoDi primarily consists of high fat intake, moderate protein consumption, and low carbohydrate intake. The macronutrient distribution often ranges from about 55 to 60% fat, 30 to 35% protein, and 5 to 10% carbohydrates. By significantly reducing carbohydrate intake and increasing fat, this diet induces ketosis, wherein the body uses fat as its primary energy source rather than carbohydrates (33). Nevertheless, it seems reasonable to state that a KetoDi should be that capable of increasing the plasma levels of KBs, independently of its carbohydrate content. This seems the case for the MeDi diet supplemented with coconut oil that we used in ALS patients.

Regarding variables and limitations of our study, although the number of participants in each group is small, and based on the SD values, both groups (healthy controls and ALS patients) were quite homogeneous and comparable (Table 1). However, all ALS patients were diagnosed with probable or definite (sporadic) ALS by El Escorial criteria, having experienced spinal symptoms for over 6 months. We did not include patients with bulbar-onset ALS, which implies a variable that could represent a different response to the KetoDi. Whether the KeDi could increase the plasma KBs in even more primary states of disease progression is another unknown variable. Furthermore, although regular physical exercise (within their possibilities) was recommended to all participants in the study, its practice was irregular, which is another variable that may influence the results.

Regarding previous findings directly related to the present study, in a preclinical study, a medium-chain triglyceride, which was metabolized into KBs, was administered to the G93A mutant SOD1 mouse model of ALS (34). This led to delayed motor function decline and better-preserved spinal cord motor neuron counts. A proof-of-concept study, also conducted in SODG93A mice, revealed that the coconut oil supplementation together with the regular diet delayed disease symptoms, enhanced motor performance, and prolonged survival (35).

In humans, a clinical trial of a ketogenic diet in ALS patients (NCT01016522, www.clinicaltrials.gov), which was terminated in 2015 due to low enrollment, the enrolled patients tolerated a KetoCal 4:1 enteral formula diet (80% fat, 17% protein, 3% carbohydrates) administered via PEG for 28 weeks. No serious adverse events occurred. After an initial 5 kg weight loss, the patients’ weight stabilized. Throughout the trial, his ALSFRS-R, pulmonary forced vital capacity, and McGill quality of life scores remained stable. In addition, a randomized, double-blind, 6-month trial compared a high-fat hypercaloric diet (8 patients) to a high-carbohydrate hypercaloric diet (6 patients) and an isocaloric diet (6 patients). Patients on the high-fat hypercaloric diet experienced more adverse events, including weight loss, than those in the other groups. Those on the high-carbohydrate hypercaloric diet had the fewest adverse events, including deaths, and were least likely to drop out of the study early (36) (NCT00983983). Furthermore, a more recent trial a randomized, placebo-controlled, multicenter trial, evaluating the effects of a high-caloric fatty diet (HCFD), revealed that the HCFD increased survival and reduced weight loss in normal to fast-progressing patients (patients with a functional decline measured by the ALSFRS-R), slowed down functional decline in the whole study population, and lowered neurofilament light chain (NfL) serum levels as a prognostic biomarker in the whole study population (NCT04172792).

## 4 Conclusion

Our results find evidence of a ketogenic effect of coconut oil in ALS patients. The facts depicted in Table 1 suggest that (a) principles derived from results obtained in healthy people may not be directly applicable to pathological conditions, particularly in diseases affecting metabolism and the way cells get energy; (b) it is feasible that metabolism in ALS patients adapts, in a time dependent fashion, to preferentially use fats.

## Data availability statement

The raw data supporting the conclusions of this article will be made available by the authors, without undue reservation.

## Ethics statement

The studies involving humans were approved by University of Valencia Institutional Review Board on Human Studies. The studies were conducted in accordance with the local legislation and institutional requirements. The participants provided their written informed consent to participate in this study.

## Author contributions

SC-J: Data curation, Formal analysis, Investigation, Methodology, Writing – review & editing. EO: Data curation, Formal analysis, Investigation, Methodology, Validation, Writing – review & editing. RL-B: Data curation, Formal analysis, Investigation, Writing – review & editing. MO-C: Data curation, Formal analysis, Investigation, Writing – review & editing. PM-M: Investigation, Methodology, Validation, Visualization, Writing – review & editing. JE: Conceptualization, Funding acquisition, Investigation, Supervision, Validation, Visualization, Writing – original draft, Writing – review & editing.

## References

[1] Carrera-Juliá S Estrela JM Zacarés M Navarro MÁ Vega-Bello MJ de la Rubia Ortí JE. Effect of the Mediterranean diet supplemented with nicotinamide riboside and pterostilbene and/or coconut oil on anthropometric variables in amyotrophic lateral sclerosis. A pilot study. Front Nutr. (2023) 10:1232184. doi: 10.3389/fnut.2023.1232184, PMID: 37810917 PMC10556480

[2] Norgren J Kåreholt I Sindi S. Is there evidence of a ketogenic effect of coconut oil? Commentary: effect of the Mediterranean diet supplemented with nicotinamide riboside and pterostilbene and/or coconut oil on anthropometric variables in amyotrophic lateral sclerosis. A pilot study. Front Nutr. (2024) 10:1333933. doi: 10.3389/fnut.2023.1333933, PMID: 38260082 PMC10801075

[3] Norgren J Sindi S Sandebring-Matton A Kåreholt I Daniilidou M Akenine U. Ketosis after intake of coconut oil and Caprylic acid-with and without glucose: a cross-over study in healthy older adults. Front Nutr. (2020) 7:40. doi: 10.3389/fnut.2020.00040, PMID: 32351966 PMC7175812

[4] Baumeister A Gardemann J Fobker M Spiegler V Fischer T. Shortterm influence of caffeine and medium-chain triglycerides on ketogenesis: a controlled double-blind intervention study. J Nutr Metab. (2021) 2021:1861567. doi: 10.1155/2021/1861567, PMID: 34221499 PMC8221889

[5] St-Pierre V Vandenberghe C Lowry CM Fortier M Castellano CA Wagner R. Plasma ketone and medium chain fatty acid response in humans consuming different medium chain triglycerides during a metabolic study day. Front Nutr. (2019) 6:46. doi: 10.3389/fnut.2019.00046, PMID: 31058159 PMC6481320

[6] Vandenberghe C St-Pierre V Pierotti T Fortier M Castellano CA Cunnane SC. Tricaprylin alone increases plasma ketone response more than coconut oil or other medium-chain triglycerides: an acute crossover study in healthy adults. Curr Dev Nutr. (2017) 1:257. doi: 10.3945/cdn.116.000257, PMID: 29955698 PMC5998344

[7] Dayrit FM. The properties of lauric acid and their significance in coconut oil. J Am Oil Chem Soc. (2014) 92:1–15. doi: 10.1007/s11746-014-2562-7

[8] Cahill GF. Fuel metabolism in starvation. Annu Rev Nutr. (2006) 26:1–22. doi: 10.1146/annurev.nutr.26.061505.111258 16848698

[9] World Medical Association. World medical association declaration of Helsinki: ethical principles for medical research involving human subjects. JAMA. (2013) 310:2191–4. doi: 10.1001/jama.2013.281053 24141714

[10] Davis C Bryan J Hodgson J Murphy K. Definition of the Mediterranean diet: a literature review. Nutrients. (2015) 7:9139–53. doi: 10.3390/nu7115459, PMID: 26556369 PMC4663587

[11] Kollewe K Mauss U Krampfl K Petri S Dengler R Mohammadi B. ALSFRS-R score and its ratio: a useful predictor for ALS-progression. J Neurol Sci. (2008) 275:69–73. doi: 10.1016/j.jns.2008.07.016, PMID: 18721928

[12] Matiegka J. The testing of physical efficiency. Am J Phys Anthropol. (1921) 4:223–30. doi: 10.1002/ajpa.1330040302

[13] Reyes ET Perurena OH Festoff BW Jorgensen R Moore WV. Insulin resistance in amyotrophic lateral sclerosis. J Neurol Sci. (1984) 63:317–24. doi: 10.1016/0022-510x(84)90154-0 6374040

[14] Pradat PF Bruneteau G Gordon PH Dupuis L Bonnefont-Rousselot D Simon D. Impaired glucose tolerance in patients with amyotrophic lateral sclerosis. Amyotroph Lateral Scler. (2010) 11:166–71. doi: 10.3109/17482960902822960, PMID: 20184518

[15] Tefera TW Steyn FJ Ngo ST Borges K. CNS glucose metabolism in amyotrophic lateral sclerosis: a therapeutic target? Cell BioSci. (2021) 11:14. doi: 10.1186/s13578-020-00511-2, PMID: 33431046 PMC7798275

[16] Tefera TW Borges K. Neuronal glucose metabolism is impaired while astrocytic TCA cycling is unaffected at symptomatic stages in the HSOD1G93A mouse model of amyotrophic lateral sclerosis. J Cereb Blood Flow Metab. (2019) 39:1710–24. doi: 10.1177/0271678X18764775, PMID: 29553298 PMC6727138

[17] Zhao Z Lange DJ Voustianiouk A Mac Grogan D Ho L Suh J. A ketogenic diet as a potential novel therapeutic intervention in amyotrophic lateral sclerosis. BMC Neurosci. (2006) 7:29. doi: 10.1186/1471-2202-7-29 16584562 PMC1488864

[18] Jang J Kim SR Lee JE Lee S Son HJ Choe W. Molecular mechanisms of neuroprotection by ketone bodies and ketogenic diet in cerebral ischemia and neurodegenerative diseases. Int J Mol Sci. (2023) 25:124. doi: 10.3390/ijms25010124 38203294 PMC10779133

[19] Dewsbury LS Lim CK Steiner GZ. The efficacy of ketogenic therapies in the clinical management of people with neurodegenerative disease: a systematic review. Adv Nutr. (2021) 12:1571–93. doi: 10.1093/advances/nmaa180, PMID: 33621313 PMC8321843

[20] Veldink JH Kalmijn S Groeneveld GJ Wunderink W Koster A de Vries JHM. Intake of polyunsaturated fatty acids and vitamin E reduces the risk of developing amyotrophic lateral sclerosis. J Neurol Neurosurg Psychiatry. (2007) 78:367–71. doi: 10.1136/jnnp.2005.083378, PMID: 16648143 PMC2077791

[21] Fitzgerald KC O’Reilly ÉJ Falcone GJ McCullough ML Park Y Kolonel LN. Dietary ω-3 polyunsaturated fatty acid intake and risk for amyotrophic lateral sclerosis. JAMA Neurol. (2014) 71:1102–10. doi: 10.1001/jamaneurol.2014.1214, PMID: 25023276 PMC4160351

[22] Maalouf M Sullivan PG Davis L Kim DY Rho JM. Ketones inhibit mitochondrial production of reactive oxygen species production following glutamate excitotoxicity by increasing NADH oxidation. Neuroscience. (2007) 145:256–64. doi: 10.1016/j.neuroscience.2006.11.065, PMID: 17240074 PMC1865572

[23] Imai SI Guarente L. It takes two to tango: NAD+ and sirtuins in aging/longevity control. NPJ Aging Mech Dis. (2016) 2:16017. doi: 10.1038/npjamd.2016.17, PMID: 28721271 PMC5514996

[24] Obrador E Salvador R Marchio P López-Blanch R Jihad-Jebbar A Rivera P. Nicotinamide riboside and Pterostilbene cooperatively delay motor neuron failure in ALS SOD1G93A mice. Mol Neurobiol. (2021) 58:1345–71. doi: 10.1007/s12035-020-02188-7, PMID: 33174130

[25] Dyńka D Kowalcze K Paziewska A. The role of ketogenic diet in the treatment of neurological diseases. Nutrients. (2022) 14:5003. doi: 10.3390/nu14235003, PMID: 36501033 PMC9739023

[26] Vargas MR Johnson DA Sirkis DW Messing A Johnson JA. Nrf2 activation in astrocytes protects against neurodegeneration in mouse models of familial amyotrophic lateral sclerosis. J Neurosci. (2008) 28:13574–81. doi: 10.1523/JNEUROSCI.4099-08.2008, PMID: 19074031 PMC2866507

[27] Paoli A Bianco A Damiani E Bosco G. Ketogenic diet in neuromuscular and neurodegenerative diseases. Biomed Res Int. (2014) 2014:474296. doi: 10.1155/2014/474296, PMID: 25101284 PMC4101992

[28] Ruskin DN Kawamura M Masino SA. Reduced pain and inflammation in juvenile and adult rats fed a ketogenic diet. PLoS One. (2009) 4:e8349. doi: 10.1371/journal.pone.0008349, PMID: 20041135 PMC2796387

[29] Feinman RD. The biochemistry of low-carbohydrate and ketogenic diets. Curr Opin Endocrinol Diabetes Obes. (2020) 27:261–8. doi: 10.1097/MED.0000000000000575 32796164

[30] Field R Field T Pourkazemi F Rooney K. Low-carbohydrate and ketogenic diets: a scoping review of neurological and inflammatory outcomes in human studies and their relevance to chronic pain. Nutr Res Rev. (2023) 36:295–319. doi: 10.1017/S0954422422000087, PMID: 35438071

[31] Bedlack R Barkhaus PE Barnes B Beauchamp M Bertorini T Bromberg MB. ALSUntangled#63: ketogenic diets. Amyotroph Lateral Scler Frontotemporal Degener. (2023) 24:159–63. doi: 10.1080/21678421.2021.1990346, PMID: 34645313

[32] Chen L Chen XW Huang X Song BL Wang Y. Regulation of glucose and lipid metabolism in health and disease. Sci China Life Sci. (2019) 62:1420–58. doi: 10.1007/s11427-019-1563-3, PMID: 31686320

[33] Kim JM. Ketogenic diet: old treatment, new beginning. Clin Neurophysiol Pract. (2017) 2:161–2. doi: 10.1016/j.cnp.2017.07.001, PMID: 30214990 PMC6123870

[34] Zhao W Varghese M Vempati P Dzhun A Cheng A Wang J. Caprylic triglyceride as a novel therapeutic approach to effectively improve the performance and attenuate the symptoms due to the motor neuron loss in ALS disease. PLoS One. (2012) 7:e49191. doi: 10.1371/journal.pone.0049191, PMID: 23145119 PMC3492315

[35] Weerasekera A Sima DM Dresselaers T Van Huffel S Van Damme P Himmelreich U. Non-invasive assessment of disease progression and neuroprotective effects of dietary coconut oil supplementation in the ALS SOD1G93A mouse model: a 1H-magnetic resonance spectroscopic study. Neuroimage Clin. (2018) 20:1092–105. doi: 10.1016/j.nicl.2018.09.011, PMID: 30368196 PMC6202692

[36] Wills AM Hubbard J Macklin EA Glass J Tandan R Simpson EP. MDA clinical research network. Hypercaloric enteral nutrition in patients with amyotrophic lateral sclerosis: a randomised, double-blind, placebo-controlled phase 2 trial. Lancet. (2014) 383:2065–72. doi: 10.1016/S0140-6736(14)60222-1, PMID: 24582471 PMC4176708

